# Dermatological Problems of Brachycephalic Dogs

**DOI:** 10.3390/ani13122016

**Published:** 2023-06-16

**Authors:** Stefan Hobi, Vanessa R. Barrs, Paweł M. Bęczkowski

**Affiliations:** 1 Department of Veterinary Clinical Sciences, Jockey Club College of Veterinary Medicine and Life Sciences, City University of Hong Kong, Kowloon Tong, Hong Kong SAR, China; vanessa.barrs@cityu.edu.hk (V.R.B.); pbeczkow@cityu.edu.hk (P.M.B.); 2 Centre for Animal Health and Welfare, Jockey Club College of Veterinary Medicine and Life Sciences, City University of Hong Kong, Kowloon Tong, Hong Kong SAR, China

**Keywords:** canine, BOAS, brachycephaly, congenital, skin folds, allergy, infectious diseases, immunologic disorders, otitis externa, ethical

## Abstract

**Simple Summary:**

Brachycephalic dogs are affected by respiratory disorders related to abnormal anatomic conformation that can significantly affect their general health and quality of life. In this review, we address dermatological disorders in these breeds which are less recognized, but can also considerably impact welfare.

**Abstract:**

Brachycephalic dogs are not only affected by brachycephalic obstructive airway syndrome (BOAS), but are also frequently referred to veterinary dermatologists for skin conditions, with English bulldogs and pugs particularly over-represented. Some skin diseases, such as skin fold dermatitis, are directly associated with the abnormal anatomic conformation of brachycephalic dogs, while for others, such as atopic dermatitis and viral pigmented plaques, there is an underlying genetic basis or a general predisposition. Anatomic alterations associated with brachycephaly, leading to fold formation of the skin and stenosis of the ear canal, together with primary immunodeficiencies described in some breeds, favor the development of pyoderma, *Malassezia* dermatitis, and otitis externa/media. In addition, the frequently neglected but often lifelong dermatological problems of brachycephalic dogs are an important consideration when discussing genetic and medical conditions affecting the welfare of those dogs. Here we review the current state of knowledge concerning dermatological problems in brachycephalic dogs and combine it with clinical experience in the management of these challenging disorders.

## 1. Introduction

Brachycephalic dogs are very popular due to cultural and social influences, as well as their “babyface” appearance and personality traits that favor bonding and companionship with their owners [1,2]. Owners may be unaware of how seriously the welfare of these breeds can be compromised by abnormalities in anatomic conformation [3,4]. Extreme brachycephaly, i.e., foreshortening of the cranium, is associated with brachycephalic obstructive airway syndrome (BOAS), leading to stridor, stertor, dyspnoea, cyanosis, exercise intolerance, regurgitation, hyperthermia, and syncope. Non-respiratory problems, including spinal, dental, gastrointestinal, ophthalmological, dermatological, and cardiovascular disorders, as well as birthing difficulties, have also been recognized [4,5]. To mitigate these problems, several countries, including the Netherlands and Norway, have instigated legal breeding restrictions, while many professional veterinary organizations, such as the British Veterinary Association, the Australian Veterinary Association, the American Veterinary Medical Association, and the Federation of European Companion Animal Veterinary Association, have launched public education awareness initiatives and campaigns [3,4,5].

The prevalence of dermatological abnormalities in brachycephalic dogs ranges from 10% to almost 30%, depending on breed and geographic origin [6,7]. Genetic, autoimmune, and parasitic diseases, immune deficiencies, vasculitis, allergies, secondary infections, otitis externa and media, claw and anal sac diseases, skin folding, alopecia, and pruritus have all been recognized as problems in brachycephalic dog breeds [8,9,10]. Genetic aspects, skull conformation, pressure changes between the middle ear and nasopharynx, skin folding, environmental factors, and the microbiome composition may all contribute to the aetiopathogenesis of dermatological diseases in brachycephalic dogs [5,11].

Many of these skin conditions may become chronic and difficult to treat, as well as causing pain and pruritus, leading to abnormal behavior and thus negatively impacting quality of life [5,12]. 

In this evidence-based narrative review, we overview the dermatological diseases encountered in brachycephalic breeds of dogs, including; (i) disorders directly associated with brachycephaly that are likely to be improved if measures to prevent extreme brachycephaly are implemented, as well as (ii) disorders that are not directly linked to brachycephalic conformation. The electronic database “Web of Science” was searched using key words including “dog”, “brachycephaly/brachycephalic”, “welfare”, “genetics”, “predisposition”, “skin diseases”, “dermatologic/dermatological”, as well as individual dog breeds (e.g., pug) and individual diseases identified during the initial search (e.g., skin fold dermatitis, otitis externa, primary secretory otitis media, etc.). Selected articles were critically appraised and compared before the incorporation of relevant information in this review.

There is no definitive list of brachycephalic breeds because no uniform measure is used. Some authors use the cephalic index (CI), the ratio of the width of the skull compared with its length, while others use the craniofacial ratio or craniofacial angle [5]. In addition, the phenotypic variation within an individual breed can be very large, such that individual dogs within a “brachycephalic breed” may not be brachycephalic, while others in non-brachycephalic breeds may indeed be brachycephalic. Table 1 lists the most commonly described brachycephalic breeds of dogs [6,13], while Table 2 lists dermatological disorders reported in brachycephalic breeds. 

## 2. Dermatological Diseases Directly Associated with Brachycephaly

### 2.1. Skin Fold Dermatitis 

Skin fold dermatitis, or intertrigo, is a major problem in brachycephalic breeds, especially in British bulldogs, French bulldogs, Pugs, Pekingese, Boston Terriers, and Shar Peis [3,9,12,14,59,60,97,98,99]. A “big-data” study that searched the medical records of 905,553 dogs presented to veterinary clinics in the UK in 2016 for skin fold dermatitis identified 11,375 cases (1.26%). Compared to cross-breed dogs, British bulldogs (odds ratio [OR] 49.07, 95% CI [37.79–63.70]), French bulldogs (OR 25.92, 95% CI [19.62–34.26]), and Pugs (OR 16.27, 95% CI [12.20–21.69]) were predisposed [12]. Foreshortening of the skull results in the folding of excessive skin around the muzzle, eyes, and ears. The problem is exacerbated in Shar Peis by increased hyaluronic acid synthetase activity, which leads to more ground substance and mucin in the dermis and attracts water [115,116]. In addition to facial involvement, skin folds can occur in other locations, such as at the tail base in dogs with “corkscrew”, such as pugs and bulldogs [12,115]. This not only leads to secondary infections but also spine instability, nerve compression, and neurological deficiencies such as pain, ataxia, and incontinence [117].

**Figure 1:**
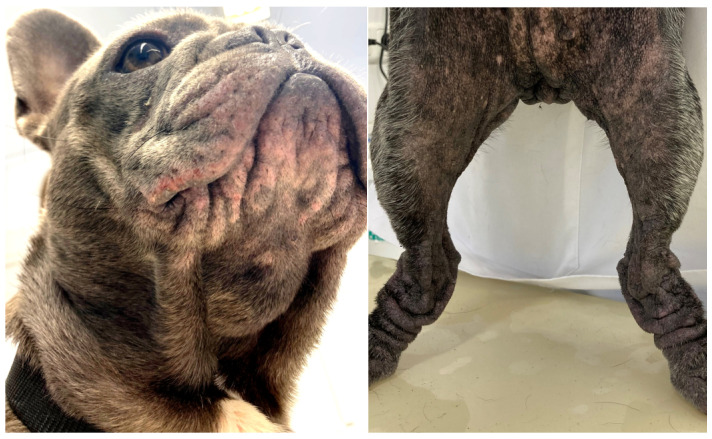
French bulldog with severe skin fold dermatitis secondary to excessive skin folds on the face/muzzle that are a direct consequence of extreme brachycephalic conformation. In addition, this dog has chronic skin fold dermatitis associated with excessive folding on the distal limbs.

Reduced air circulation and increased temperature, humidity, and debris within skin folds, together with intermittent friction and trauma, lead to commensal overgrowth and toxin production, followed by inflammation, maceration, and infection [14,115]. Affected areas exhibit erythema, hypotrichosis, alopecia, erosion/ulceration, crusting, lichenification, pigmentary changes, accumulation of keratosebaceous debris, and malodour (Figure 1). 

Involved areas can be pruritic and painful. Since the changes take place between skin folds, disease may not be noticed by owners [12,14,115]. If corrective surgery is not an option, lifelong treatment may be required with various topical preparations (e.g., antiseptics, glucocorticoids, antimicrobials, medical honey, or silver sulfadiazine). In severe cases where there is deep pyoderma, systemic antimicrobial therapy may be indicated and used according to culture and susceptibility test results [12,14,115].

### 2.2. Otitis Externa

Otitis externa (OE), inflammation of the ear canal and often the outer ear, is more prevalent in brachycephalic dogs, especially British bulldogs, Pugs, and Boxers, than in non-brachycephalic dogs [8,59,60,69]. Otitis externa is associated with predisposing factors (e.g., anatomic conformation, swimming), primary factors (direct induction of inflammation, e.g., parasites, food allergy, atopy, foreign body, growths, hormonal), secondary factors (e.g., secondary infection by commensals), and perpetuating factors (chronic changes of the ear canal, ear drum, or middle ear). Recently, it was shown that two brachycephalic breeds of dogs, French bulldogs and Pugs, have significantly narrower external ear canals than non-brachycephalic dogs of similar size [118]. The diameter of the horizonal ear canal was measured between its cranial and caudal bony walls on computer tomographic (CT) scan images and had a median value of 2.5 mm, 2.6 mm, and 5.0 mm in French bulldogs, Pugs and non-brachycephalic control dogs, respectively. In addition, on otoscopic examination, the tympanic membrane could only be visualized in 3.3% of brachycephalic dogs due to ear canal stenosis. Among the brachycephalic dogs examined in the study, no significant association was made between the presence of OE and ear canal diameter. However, their striking differences in ear canal diameter compared to non-brachycephalic dogs suggest that OE is likely a direct consequence of brachycephalic conformation, at least in some cases. Other predisposing factors to OE, including allergic skin diseases, are discussed in Section 3.4.2. 

**Figure 2:**
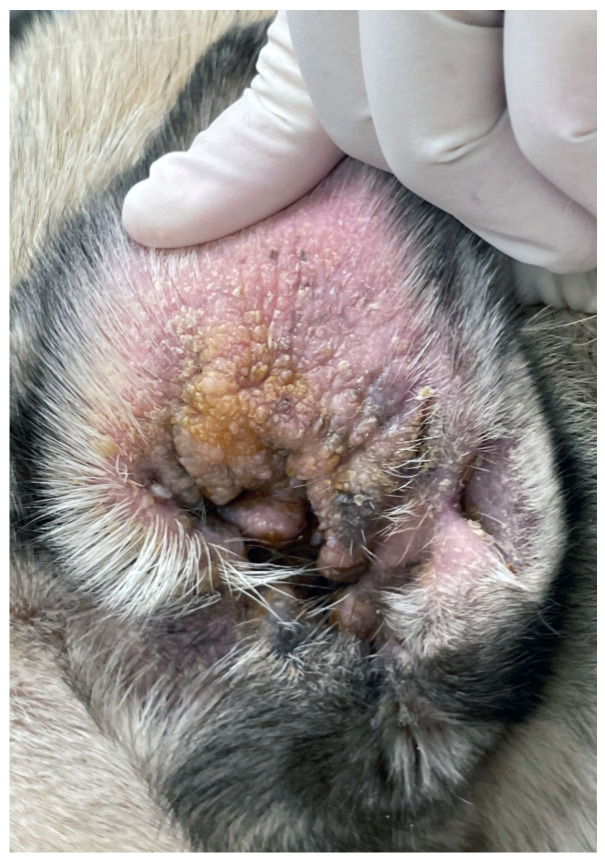
Chronic otitis externa in a Pug showing erythema, lichenification, crusting and accumulation of keratosebaceous debris.

Clinical signs of OE include abnormal scratching of the pinnae, excoriations, head shaking, otic discharge, malodour, swelling, pain, formation of “hot-spots” (moist dermatitis), and othematoma (Figure 2) [14,119]. If left untreated, OE may further progress to involve the middle ear (otitis media), the internal ear (otitis interna), and extend into the central nervous system (CNS). Diagnosis is usually made by otoscopy and cytology, but advanced investigations (video-otoscopy, CT/MRI) may also be required [119,120]. 

Treatment of OE typically includes a combination of topical ear drops, an ear cleaner, and, if not contra-indicated, an anti-inflammatory dose of oral glucocorticoids to reduce the stenosis, pruritus, and pain [14,121]. It is widely recognized by veterinary dermatologists that OE is painful, and consideration should be given to dispensing analgesics in all cases. Evidence-based recommendations are lacking and highlight the need for studies to evaluate and manage pain in dogs with OE. Flushing of the ear canal under anaesthesia can help to remove debris, toxins, biofilm, and exudates, but it also increases the efficacy of topical medications [14,119,120]. Biofilm can also be disrupted by topical silver nanoparticles, Tris-EDTA, and oral n-acetylcysteine or bromhexine [121,122,123]. Topical ear preparations may be ototoxic (e.g., macrolide, polypeptide, and aminoglycoside antibiotics, propylene glycol, ceruminolytics, and antiseptics) and must be used cautiously. Ototoxicity can lead to hearing loss, imbalance, or nausea by direct effect on the hair cells, stria vascularis, or cochlear nerve of the internal ear or via the formation of reactive oxygen species [124,125].

### 2.3. Caudal Occipital Malformation Syndrome/Chiari-like Malformation

This congenital and multifactorial inherited abnormality was first recognized and reported in Cavalier King Charles Spaniels (CKCS), with up to 95% of individuals being affected [36,37,38,39]. It is also recognized in other brachycephalic small-breed dogs [40,41,42,43]. The caudal occiput is too small-relative to the cerebellum, which may prolapse through the foramen magnum, leading to an abnormal flow of cerebrospinal fluid and the formation of a fluid-filled cyst (syrinx) within the spinal cord (syringomyelia). In chronic cases, spinal cord degeneration, including ventral horn cell or white matter damage, may complicate the situation [126,127]. Neuropathic pain results in “air-guitar” scratching, “pseudo-fly catching”, spontaneous vocalization and hopping, repeated body shaking, and severe rubbing of the face on the floor [127,128]. In more severely affected dogs, other signs may be present, including ataxia, head tilt, head tremor, facial nerve deficits, nystagmus, seizures, and scoliosis [43,127]. 

Magnetic resonance imaging (MRI) is the modality of choice to diagnose Chiari-like malformations, but computed tomography (CT) may also be used [129].

Medical treatment with non-steroidal inflammatory inhibitors, glucocorticoids, opioids, and anticonvulsants (gabapentin and pregabalin) helps to relieve pain, while omeprazole, acetazolamide, and methazolamide may be prescribed to reduce the formation of cerebrospinal fluid [130,131]. Alternative pain management options such as acupuncture and laser therapy are becoming more popular and may help as well, but progressive disease is common and surgical intervention may be required in severe cases [128,132]. For severe cases or dogs not responding to medical treatment, there is up to an 80% chance of clinical improvement following foramen magnum decompression and durotomy [43,133]. Duraplasty or craniotomy/cranioplasty in combination with tissue grafting/titanium prosthesis/titanium mesh/polymethylmethacrylate plate further improves the success rate [129,134]. However, despite surgical intervention, residual scratching is often reported [129,135].

### 2.4. Primary Secretory Otitis Media/Otitis Media with Effusion

Primary secretory otitis media, a sterile effusion of the middle ear, is mainly described in CKCS but also in other brachycephalic breeds such as Boxers and Boston Terriers [44,45,46,136]. In CKCS, around 40% may concurrently have Chiari-like malformations [45]. Auditory tube dysfunction, associated with craniofacial abnormalities, is implicated in disease pathogenesis [137,138]. 

Affected dogs present with head and neck pain, spontaneous vocalization, abnormal neck carriage, fatigue, aural pruritus, neurological signs (facial nerve paralysis, nystagmus, head tilt, ataxia, seizures), or reduced hearing, but may also be asymptomatic [44,45].

Diagnostic work-up includes video otoscopy (bulging, opaque pars flaccida of the tympanic membrane; can be normal in Boston Terriers), myringotomy (thick, mucoid discharge), and MRI or CT [45,136]. For an accurate diagnosis and prognosis, advanced imaging is preferred. Other conditions, such as CLM or cholesteatomas, the latter being commonly seen in French Bulldogs and Pugs, need to be excluded [136,139]. Although most of the time sterile, the secret of the middle ear should be cultured since bacterial involvement is possible [44]. 

Myringotomy of the pars tensa of the tympanic membrane with subsequent flushing of the bulla, systemic antibiotics, topical ear drops, and a short course of systemic anti-inflammatory glucocorticoids are common treatment steps [44,45,46]. Additional mucolytic N-acetylcysteine may be beneficial. Relapses, one to several months after treatment, are possible [45].

## 3. Skin Diseases in Brachycephalic Breeds That Are Not Directly Linked with Brachycephalic Conformation

### 3.1. Genetic Skin Diseases

#### 3.1.1. Ichthyosis

Ichthyosis is a rare genetic disease affecting various breeds, including CKCS and American Bulldogs [29,30,31,32,33]. In the latter, a mutation in NIPAL-4 (nipa-like domain-containing 4, ICHTHYIN) is implicated in abnormal lipid metabolism in the epidermis [30]. In a multicentric study, approximately 35% of tested dogs were heterozygote carriers, and 5.4% were clinically affected. The disease was associated with an autosomal recessive insertion mutation 5781 bp upstream of NIPAL-4 [30]. Fine scaling throughout a rough hair coat, prominent erythematous to brown scales on the axillae and abdomen, together with wrinkling of the skin, are typical features described in affected American bulldogs. Secondary *Malassezia* dermatitis/overgrowth, pododermatitis, and otitis externa are common sequela [30,140]. 

In CKCS, the condition is caused by a mutation in FAM83H (family with sequence similarity 83, member H), which is yet to be further characterized [140]. In CKCS, a roughened, scaly, and curly haircoat, together with a hyperpigmented abdomen, footpad hyperkeratosis, and nail abnormalities (nail dystrophy, onychomadesis), become apparent. Affected dogs also have keratoconjunctivitis sicca and may become blind if this goes undetected [29]. 

In both breeds, the first clinical signs occur directly after birth [140]. A definitive diagnosis can be obtained via histopathology or genetic blood testing in cases of ichthyosis in American bulldogs [30,140]. Since ichthyosis is a congenital disease, only symptomatic treatment, including treatment of secondary infections, regular combing, mild shampoo treatment, systemic and topical fatty acids, as well as systemic retinoids (isotretinoin, etretinate), can be employed [14,140]. Affected dogs should not be used for breeding. 

#### 3.1.2. Congenital Alopecia 

Congenital alopecia is a rarely observed problem in various brachycephalic and other canine breeds, including the French bulldog, Lhasa Apso, and Chihuahua [14,15,16,17]. It typically occurs within weeks to months after birth and is associated with an x-linked, autosomal dominant, or autosomal recessive trait [14,15,24]. The disease phenotype ranges from hypotrichosis to alopecia, which may be localized or generalized [14,15]. Hair loss is typically well-demarcated, occurring on the head, ears, and ventrum [14,15]. Some residual hair, symmetrically arranged, can be observed on the dorsal head, distal limbs, tail, umbilical area, and around mucocutaneous sites [15]. In more chronic cases, scaling and hyperpigmentation may occur [14]. This needs to be differentiated from ectodermal dysplasia, where other structures such as sweat glands, sebaceous glands, respiratory glands, lacrimal glands, claws, and teeth are involved as well [141]. A definitive diagnosis of congenital alopecia is made through the collection of multiple skin biopsies from different skin sites that exhibit a complete absence or a decreased number of hair follicles [14,15]. There is no specific treatment. Prevention can be effectively achieved by avoiding the breeding of affected individuals [14]. 

#### 3.1.3. Colour Dilution Alopecia (CDA)/Black Hair Follicular Dysplasia/Follicular Dysplasia (FD)

These dermatopathies are reported in both brachycephalic and non-brachycephalic dog breeds, including Chihuahuas, Yorkshire Terriers, Shih Tzus, Boxers, Boston Terriers, Cavalier King Charles Spaniels, and blue Chow Chows [14,18,19,20,21,22,23]. The disease is inherited by an autosomal-recessive trait with singular or multiple mutations within or near the melanophilin gene [142,143]. Melanin precursors with cytotoxic effects and abnormal pigment clumps in the epidermis, hair shaft, hair follicle, and hair matrix lead to bulging and fracture of the hair cuticle and therefore alopecia [144]. Progressive hypotrichosis, alopecia, and scaling develop in the affected areas. In color dilution alopecia (CDA), there is also folliculitis and furunculosis. The full extent of disease is usually recognized around 2 to 3 years of age, or earlier in the case of follicular dysplasia [14,18,144]. 

An increased risk for cancer development has been described for CDA [145]. Trichograms, showing numerous macromelanosomes within the hair shaft leading to irregularities and distortion, and skin biopsies with histopathology exhibiting dilated hair follicles filled with keratin, hair shafts, free melanin, and abnormal melanin clumps in the epidermis and hair follicles, are important diagnostic tools. Commercially available DNA tests targeting the Ras-related protein Rab-27 (RAB27) or malanophilin (MLPH) are now also available [14,18,142,144]. There is no specific treatment, and trauma as well as intense UV-light exposure should be avoided [14,18,144]. Oral retinoic acid may be beneficial [14].

#### 3.1.4. Canine Flank Alopecia/Seasonal Flank Alopecia

**Figure 3:**
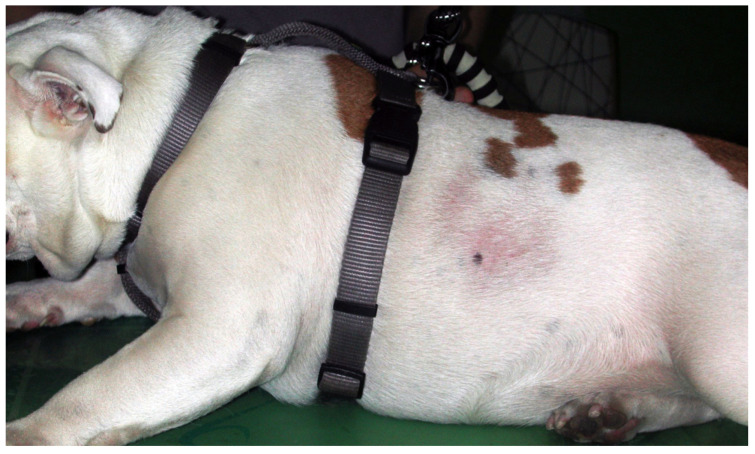
English Bulldog with seasonal flank alopecia.

This localized, cyclic, likely polygenetic follicular dysplasia has a high prevalence in middle-aged Boxers and Affenpinschers but is also reported in other breeds, including English bulldogs, Chihuahua, and Staffordshire Bull Terrier [25,26,27,146]. The aetiology is not known, but reduced light exposure and an association with melatonin are considered likely [14,147]. Well-circumscribed, non-pruritic, hyperpigmented, mostly symmetric alopecia, forming a geographic map appearance, typically develops over the flanks during winter (Figure 3). Spontaneous hair regrowth, which may be associated with color change, occurs within 1 to 14 months. Occasionally, alopecia becomes permanent [14,146,148]. Around 20% of individuals only have one episode, whereas most dogs have recurrent alopecic episodes in the following years [146,148]. Affected individuals are otherwise healthy. The breed, history, clinical signs, and exclusion of endocrinopathies make a diagnosis very likely, but in atypical cases, histopathology may be warranted. Since this is a cosmetic problem, observation without treatment is an option, but affected individuals should not be used for breeding. Treatment success can be achieved with melatonin (oral and implanted) and increased contact with the sun/artificial light [14,146,147,148]. 

#### 3.1.5. Pattern Baldness

Canine pattern baldness is an uncommon disease occurring in both brachycephalic and non-brachycephalic breeds [14,28]. Four different syndromes have been described in Dachshunds, another in American Water Spaniels, a third in Greyhounds, and a fourth syndrome in various breeds, including English bulldogs, Boston Terriers, Boxers, and Chihuahuas [14,28].

Disease often starts around 6 to 9 months of age and progresses over months or years [14,28]. The cause is not known, and an association with androgen receptor dysfunction, as has been described in humans, could not be shown [28]. The fourth syndrome is most common, especially in female dogs, and affects the periauricular skin, the ventrum, the perineal region, and the caudomedial thighs [14,28]. Affected areas do not show complete hair loss but rather miniaturized hair [14,28]. In chronic cases, hyperpigmentation and scaling may occur [14]. A trichogram can help confirm a diagnosis if the patient has normal hair in non-affected areas and miniaturized hair in affected areas. Histopathologically, hair follicles and hair shafts are smaller and thinner than normal [28]. Due to the cosmetic nature of this disease, treatment is not necessary, but oral melatonin may be beneficial [149]. If successful, improvement is typically seen within around 6 weeks [149].

#### 3.1.6. Tyrosinase Deficiency

This genetic abnormality is rarely seen in Chow Chow puppies [14,35]. Affected dogs have a pink (instead of black) tongue, depigmentation of the buccal mucosa, and whitening of the haircoat. They are otherwise healthy [14,35]. Since tyrosinase is necessary to produce melanin, supplementation of tyrosinase to histopathologic preparations and melanin measurement after tissue staining can help with the diagnosis [14,35]. There is no specific treatment, but due to the spontaneous reappearance of melanin, improvement is seen within 2 to 4 months [14,35].

#### 3.1.7. Cutaneous Asthenia

This rare genetic disease occurs in various canine breeds, among which boxers are more frequently affected [14,34]. Both autosomal-recessive and dominant genetic mutations are reported [14,34]. The skin is thin, hyperextensible, and can be easily torn, leaving “fish-mouth” ulcerated wounds that have minimal to no bleeding and heal quickly to leave characteristic “cigarette-paper”-like scars. Rarely, other manifestations such as widening of the bridge of the nose, inguinal and umbilical hernias, increased joint laxity, hygroma formation, and ocular changes can occur [14]. Cutaneous asthenia is associated with an increased skin fragility index, i.e., the distance between the occiput and the base of the tail divided by the length of a stretched skin fold from base to top (>14.5%) [150]. Histopathology classically shows abnormally arranged, irregular collagen fibers with atypical staining properties (Masson trichrome stain). These changes are not always visible and clear [151,152]. 

Since vitamin C is involved in collagen synthesis, oral supplementation may be beneficial [14]. Lifestyle and housing adjustments are needed to reduce the chance of trauma and wound formation. Such measures include soft bedding, the removal of sharp corners and rough surfaces, and reduced interactions with other animals [14]. One of the authors (SH) has successfully used special protective body suits. Affected animals should not be used for breeding [14].

### 3.2. Infectious Skin Diseases

#### 3.2.1. Canine Demodicosis

Canine demodicosis is a common parasitic disease that can occur at a young age or later in life [10,153]. Adult-onset demodicosis is typically associated with an underlying disease (hormonal, neoplasia, or immunosuppression) [10,153]. Juvenile disease is the result of a mostly temporary immune alteration, leading to an overgrowth of these commensal mites [10,153,154]. Other predisposing factors include inadequate nutrition, severe stress, parturition, and post-partum oestrus and endoparasites [155,156]. Many brachycephalic breeds, including Pugs, Boxers, English bulldogs, French bulldogs, Shih Tzus, Chow Chows, Boston Terriers, Staffordshire Bull Terriers, Shar Peis, and Chihuahuas, are predisposed [10,47,48,49,50,51,52,53,54,55,56].

Various degrees of multifocal hypotrichosis, including alopecia, erythema, crusts, scales, follicular casts, papules, pustules, nodules, hyperpigmentation, lichenification, and comedones, occur on the head, trunk, limbs, and paws [10,153]. Secondary infections, especially with bacteria, are common and may lead to a mild degree of pruritus [48,153]. Ceruminous otitis externa can also be seen [157]. In severe cases, especially if immunosuppressed and left untreated, deep bacterial infections can lead to sepsis and unspecific systemic signs like fever, anorexia, lethargy, and peripheral lymphadenopathy [48]. 

Different stages of demodex mites (larvae, adults, and eggs) can be identified via a deep skin scrape, trichogram, or acetate tape squeezing technique [153]. Depending on the severity of the presentation, the general condition of the patient, and the form of demodicosis, active surveillance is sufficient, while medical treatment may be initiated in selected cases [48,153]. For the juvenile form, even with generalized disease, spontaneous remission is reported [153]. Additionally, since there is a genetic predisposition for juvenile-onset disease, breeding affected individuals is not recommended [153,158]. Desexing of affected intact female dogs is recommended due to flare-ups during oestrus [159]. In adult-onset disease, correction of the underlying cause is indicated [160]. Amitraz, macrocyclic lactones, and isoxazolines are efficacious, but potential adverse effects and drug legislation should be considered when selecting these drugs [47,153,160]. 

#### 3.2.2. *Malassezia* Dermatitis

A nationwide insurance analysis in the US recognized an increased risk in brachycephalic dogs for fungal skin diseases [161]. *Malassezia* spp. are yeasts and are skin and mucosal commensals [162]. This fungal organism is commonly associated with dermatitis, including intertrigo, otitis externa, paronychia, and rarely keratomycosis [162]. Brachycephalic breeds predisposed to *Malassezia* dermatitis include the Shih Tzu, English bulldog, Boxer, Cavalier King Charles Spaniel, and Lhasa Apso [14,57,58]. 

*Malassezia* dermatitis can cause hypotrichosis, alopecia, erythema, scales, crusts, greasiness, lichenification, hyperpigmentation, and variable pruritus, especially on the concave pinnae, muzzle, ventral neck, perianal, medial thighs, axillae, inguinal, and paws [162]. Typical triggers include hypersensitivities (flea bite hypersensitivity, food allergy, atopic dermatitis), ectoparasites, superficial pyoderma, endocrinopathies, keratinization abnormalities, and autoimmune diseases [162]. Diagnosis can easily be achieved via cytological examination of affected areas, showing round to oval to peanut-shaped organisms of 3 to 8 µm [14]. Apart from addressing the underlying cause, topical treatment with chlorhexidine or azole preparations is preferred, and systemic therapy with itraconazole, terbinafine, or fluconazole should be reserved for severe, generalized cases or where topical treatment fails [162,163].

#### 3.2.3. Viral Pigmented Plaques

**Figure 4:**
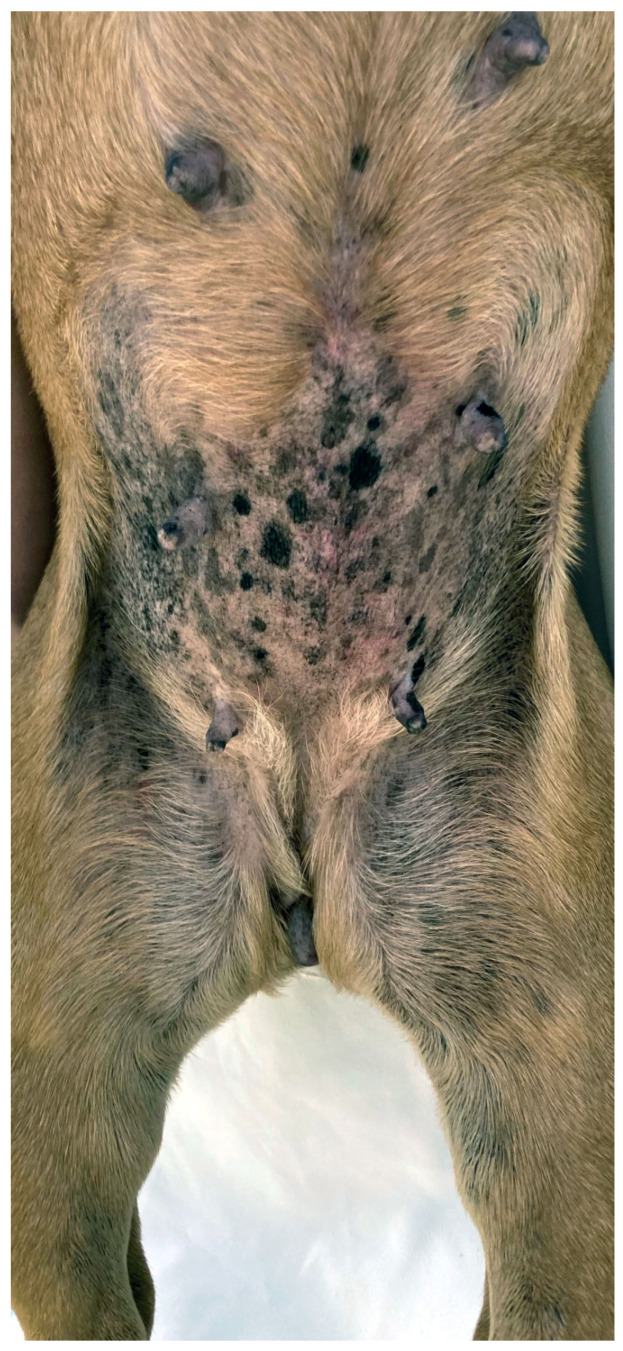
Pug with multiple viral plaques caused by *Chipapapillomavirus* infection.

This viral skin disease associated with *Chipapillomavirus* is reported in many brachycephalic breeds, including the Pug, French bulldog, Chihuahua, and Boston Terrier, as well as in non-brachycephalic breeds [65,66,67,68]. The onset of the disease may be related to a genetic immunodeficiency, as reported in pugs, Vizslas, and Chihuahuas, or secondary to immunosuppression [164]. Numerous small, plaque-like, hyperpigmented lesions with an irregular and scaly surface appear on the ventral neck, thorax, abdomen, and ventro-medial, proximal limbs (Figure 4). A progression to wart-like lesions is described [164]. Depending on the location and number of lesions, discomfort and pruritus can occur [164]. Lesions further progress, especially at the beginning, and rarely transform into squamous cell carcinoma [165]. The clinical appearance together with histopathology often allow a diagnosis, but in the early stages of the disease, further workup, such as PCR, may be needed [166]. Several treatment options are described, including surgical removal, laser treatment, cryotherapy, systemic azithromycin, interferons, and retinoids, as well as topical agents such as vitamin A, imiquimod, or tigilanol tiglate gel [164,166].

### 3.3. Bacterial Skin Diseases

#### 3.3.1. Bacterial Folliculitis (Superficial Pyoderma)

Brachycephalic breeds are predisposed to bacterial skin infections, as indicated by an insurance survey in the US as well as an Australian study [3]. The British Bulldog, Pug, Boxer, Shar Pei, and Bullmastiff are predisposed to superficial bacterial folliculitis, which is usually associated with *Staphylococcus pseudintermedius* [9,14,59,60]. Clinical signs range from mild (loss of hair gloss, increased shedding, erect hairs, or mild scaling) to severe (alopecia, erythema, follicular papules/pustules, epidermal collarettes, and crusts). This may lead to secondary pruritus and deep pyoderma [14,167]. Common underlying triggers are allergies, trauma, ectoparasites, dermatophytes, excessive brushing, seborrhoea, and systemic diseases [14,168]. Diagnosis can be made by recognition of characteristic lesions, cytology (presence of cocci and inflammation), culture, and susceptibility testing [14,169]. Topical treatment with products containing chlorhexidine, benzoyl peroxide, or ethyl lactate is recommended. Systemic antimicrobial therapy is reserved for widespread, deep pyoderma or where topical treatment alone fails [14,168]. 

#### 3.3.2. Pyotraumatic Dermatitis (Hot Spot)

This skin condition is characterized by a peracute onset of severe pruritus associated with a well-demarcated area of alopecia, erythema, swelling, papules, pustules, and crusts. British bulldogs, Pugs, and Rottweilers are predisposed [9,59,60,61,97].

#### 3.3.3. Muzzle Folliculitis and Furunculosis

Muzzle folliculitis and furunculosis, another form of bacterial infection restricted to the skin of the muzzle, present with pruritus, alopecia, erythema, swelling, papules, pustules, erosion/ulceration, crust formation, and haemorrhagic bullae. An increased risk is recognized in the British Bulldog, Boxer, Rottweiler, and brachycephalic breeds overall [3,14,62].

#### 3.3.4. Canine Leproid Granuloma

Boxers are predisposed to this infectious disease, suggesting a genetic predisposition [14,63,64]. Disease, caused by mycobacterial strains of the *Mycobacterium simiae* clade in association with trauma, previous skin lesions, and insect bites, is most prevalent in Australia, the USA, and South America (Brazil) [170]. Affected individuals show multiple, intact to ulcerated, well-demarcated nodules and plaques on the head (especially pinnae) and limbs but are otherwise healthy [170]. The diagnosis is based on clinical, cytological (acid-fast bacilli), and histopathological findings [170]. Although there is a chance for spontaneous remission within one to three months, systemic treatment with azithromycin and rifampicin with or without surgery may be needed, particularly in more severe and refractory cases [170]. Topical formulations may be supportive [171].

### 3.4. Immunological Skin Diseases

#### 3.4.1. Hypersensitivities

Many brachycephalic breeds show an increased risk for different forms of allergy, including flea bite hypersensitivity (FBH; Chow Chow), food allergy (FA; Lhasa Apso, Boxer, Shar Pei), and atopic dermatitis (AD; Boxer, American bulldog, English bulldog, French bulldog, Boston Terrier, Lhasa Apso, Shih Tzu, Chow Chow, Pug, Staffordshire Bull Terrier, Shar Pei) [14,75,76,77,78,79,80,81,82,83,84]. The pathogenesis of most of these diseases is complex and still not fully understood, but likely includes a combination of genetic, skin/mucosal barrier, immunologic, and skin/mucosal microbiome abnormalities [11]. All of these conditions are characterized by variable primary pruritus associated secondary lesions and are further complicated by secondary bacterial and yeast infections (otitis externa, *Malassezia* dermatitis, pyoderma, pododermatitis/furunculosis). 

**Figure 5:**
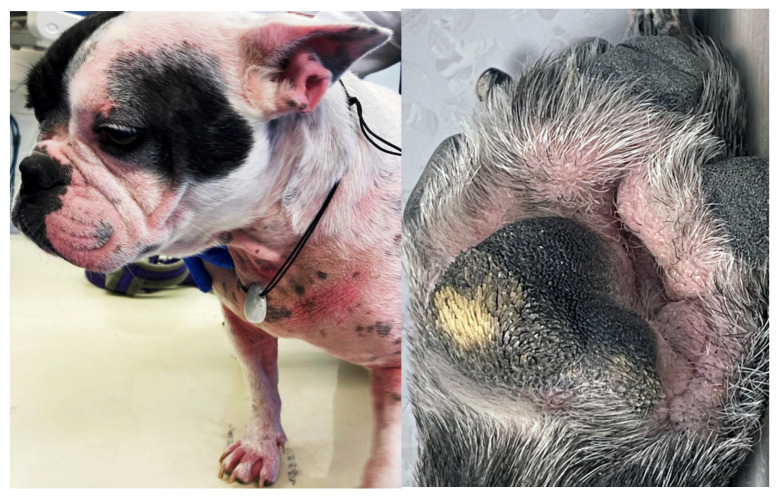
Atopic French Bulldog with chronic allergic dermatitis, including mild to moderate alopecia, erythema, lichenification, and accumulation of keratosebaceous debris on the pinnae, muzzle, ventral neck, chest, dorsal elbows and paws. Fold formation as a consequence of brachycephaly as well as abnormal wear of the paw pads negatively influence allergic disease.

In many brachycephalic breeds, especially Pugs and French bulldogs, the nails and footpads do not wear down normally, further contributing to and worsening allergic pododermatitis [172,173]. Primary pruritus mainly affects the posterior in FBH, whereas in FA and AD the ears, face, muzzle, ventral neck, distal limbs, paws, axillae, inguinal, and perineum are commonly affected (Figure 5) [82,174,175,176,177]. Atopic dogs and dogs with FA may also present with anal sac impaction, acute moist dermatitis, acral lick dermatitis, seborrhoea, hyperhidrosis, rhinitis, reverse sneezing, gastrointestinal disturbances, and sexual cycle abnormalities [178]. Alternatively, dogs with FA may have other presentations such as erythema multiforme, cutaneous vasculitis, urticaria, anaphylaxis, seizures, and behavioral changes [14,179]. 

Diagnosis of the different forms of allergy can be achieved by a response to flea treatment, a strict elimination diet over 4 to 8 weeks with subsequent provocation, the exclusion of other causes of pruritus, and the application of specific established criteria (Favrot’s criteria) [11]. Dogs with FBH or FA can be managed with the use of appropriate flea control and/or dietary interventions [14]. A multimodal approach is often required for the treatment of AD, including addressing the pruritus, secondary infections, and skin barrier, especially if allergen-specific immunotherapy is insufficient [11,14]. 

#### 3.4.2. Pemphigus Foliaceus

Pemphigus foliaceus is the most common canine autoimmune skin disease that mainly occurs in middle-aged and older animals [14,85]. Multiple breeds can be affected, but Chow Chows have an increased risk [85,86,87,88,89]. Several factors, including genetics, drugs, insects, UV-light, and chronic inflammation, may trigger an autoimmune response targeting desmocollin-1, leading to acantholysis and pustule formation [180]. The disease mainly affects the pinnae, dorsal nose, and paws but may progress to involve other sites. The distribution is often symmetrical, and affected dogs show transient papules and pustules, intense crusting, alopecia, epidermal collarettes, and fissures on the paw pads. There is variable pruritus and secondary bacterial and *Malassezia* infections. In severe cases, fever, lethargy, anorexia, and lymphadenopathy are also present [85,181]. Cytology of intact pustules reveals neutrophils, eosinophils, and acantholytic cells in the absence of bacteria. Since acantholytic cells can also occur with fungal (*Trichophyton* spp.) and bacterial infections (*Staphylococcus* spp.), these organisms must be excluded [14,85]. A definitive diagnosis is attained via multiple skin biopsies and histopathology [85]. Treatment typically includes topical and systemic antimicrobials as well as immunosuppressive drugs such as glucocorticoids, cyclosporine, azathioprine, chlorambucil, mycophenolate mofetil, and recently, oclacitinib [14,85,181]. Potential triggers should be eliminated. Cases with vascular involvement may show more serious clinical signs, be more challenging to treat, and take longer to achieve remission [181]. Most patients require life-long treatment, and few die or will be euthanized due to treatment failure, drug side effects, complications, and/or lack of compliance [85,181].

#### 3.4.3. Uveodermatologic Syndrome

This rare immune-mediated disease primarily affects Akitas but also occurs in other breeds, including Chow Chows [14,90,91]. The pathogenesis is complex, including a heritable component (canine leukocyte antigen alleles) and an inflammatory response including Th17, Th1, and Th2 helper cells with the formation of associated cytokines, autoantibodies, and macrophages infiltration, targeting pigmented structures of the eyes, ears, hair, skin, and nervous system [14,182]. The disease occurs in young to middle-aged dogs, presenting as bilateral photophobia, blepharospasm, epiphora, and blindness. Skin abnormalities classically occur later on, are bilaterally symmetric, and show depigmentation, leukotrichia, leukoderma, alopecia, erythema, scaling, erosion/ulceration, crusting, hyperkeratosis, and rarely onychomadesis or swelling of the nose. The nasal planum, periocular skin, lips, oral cavity, genitals, and footpads are commonly involved [183,184]. Neurologic and auditory signs are rarely reported, might be very subtle, and are thereby underdiagnosed [183]. A rapid diagnosis is very important to avoid blindness. It includes a complete ophthalmological examination and histopathology in cases of skin involvement [183,184]. Ophthalmic glucocorticoids, together with oral immunosuppressive doses of glucocorticoids, are indicated. Initial treatment can be enhanced by the addition of systemic cyclosporine, azathioprine, or other steroid-sparing immunosuppressants in refractory cases [14,184]. 

#### 3.4.4. Sterile Granuloma and Pyogranuloma Syndrome

Boxers, English bulldogs, and French Mastiffs are predisposed to this rare immune-mediated disease [14,96]. Infectious (bacteria, fungi, parasites, and protozoa) and foreign bodies must first be ruled out before inflammation can be considered sterile [185]. Usually, there are multiple lesions consisting of non-pruritic, non-painful, erythematous, haired to alopecic, often ulcerated, fistulated, and crusted, papules to nodules and plaques, especially occurring on the head and distal limbs. The lesions can spontaneously resolve but also wax and wane [185,186]. Definitive diagnosis requires bacterial and fungal culture, histopathology, including a variety of special stains, and ideally also Leishmania and mycobacterial PCR testing [185,186]. Control can be achieved by immunosuppressive drugs, including glucocorticoids, azathioprine, and cyclosporine. Oral fatty acids may have beneficial or drug-sparing effects. Tetracycline/doxycycline together with niacinamide may also be beneficial in selected cases, but as part of good antimicrobial stewardship, this approach should be avoided where possible [14,187].

#### 3.4.5. Acute Febrile Vasculitis

This vascular disease is rare but is exclusively seen in Shar Peis [92,93,94,95]. The cause of it is not known, but vaccines, insect bites, and infectious microorganisms are discussed as potential triggers [92]. The disease occurs in young puppies, with affected individuals showing acute fever, lethargy, anorexia, lymphadenopathy, and dramatic skin changes comprising severe swelling, well-demarcated ulceration, and necrosis, as well as haemorrhagic maculae, vesicles, and bullae on the head, limbs, and trunk [92]. Diascopy is an easy, cheap, and fast test to recognize bleeding into the skin, but further workup involving comprehensive blood tests, imaging, and skin biopsies is usually warranted [92]. The described treatments include wound and pain management, immunosuppressive and antimicrobial therapy, and surgery. Potential triggers should be eliminated and avoided. The prognosis is guarded, with some affected individuals succumbing to the disease despite treatment [92,95]. 

#### 3.4.6. Primary Immune Deficiencies 

Very rarely, dogs are born with specific immune deficiencies, leading to recurrent infections of the skin, respiratory, urogenital, and/or gastrointestinal tract. These deficiencies include cyclic haematopoiesis (Pomeranian), T-cell dysfunction (Bull Terrier), IgA/IgG (Chow Chow, Rottweiler), and granulocyte colony stimulating factor (G-CSF) (Rottweiler) abnormalities. Affected individuals are young, and the skin might be affected by juvenile demodicosis, recurrent secondary pyoderma, and subcutaneous abscesses [14,70,71,72,73,74].

### 3.5. Miscellaneous Skin Diseases

#### 3.5.1. Anal Sac Disease

Anal sac disease is common in dogs overall but is especially common in brachycephalic dogs (up to 2.62 times the odds of dolichocephalics), particularly Pugs (up to 2.23 times the odds of non-Pugs) [3,6,97,100,188]. Obesity, soft stools, intestinal disorders, changes in muscle tone, and relatively small anal sac ducts are contributing factors to disease [178]. Recurrent anal sac disease is often associated with AD or FA [14,178]. Anal sac impaction may progress to sacculitis and abscess formation. Perianal pruritus, tail chasing, scooting, tenesmus, and abscess formation are common reasons for presentation. Clinical signs, digital palpation, and perianal evaluation help with diagnosing these problems [14,178]. Anal sacs can be expressed, lavaged, topical antimicrobials instilled, or, in more severe cases, systemic antibiotics, wound treatment, and surgical excision considered. In cases of sacculitis, cannulation and flushing of the anal sac with normal saline, 0.025% chlorhexidine, or 0.4% povidone-iodine solution can be done via 22 to 24 G catheter. Often, a commercially available steroid/antifungal/antibiotic solution/ointment is instilled thereafter [189]. Since topical treatment is often effective, systemic antibiotics should only be used in refractory or severe cases [190]. In addition, underlying problems should be identified and corrected [14,178]. 

#### 3.5.2. Pododermatitis and Furunculosis

This is a common and often frustrating disease complex affecting the ventral, dorsal-interdigital, pad, and/or ungual parts of the paw [14,191,192]. A predisposition to this disease is described in brachycephalic breeds, particularly English bulldogs, Staffordshire Bull Terriers, and Boxers [6,9,14,60,191,193]. Disease pathogenesis is associated with abrasive short hair coats and abnormal footpad wear, resulting in external trauma to the hair follicles of the palmar and plantar webs, leading to cyst formation, cyst rupture, foreign body reaction, fistulation, and secondary bacterial infection [192,194]. 

In the majority of cases, more than one foot is affected [192]. Atopy and food hypersensitivity are the most common underlying causes, followed by podo-demodicosis. Other causes include infections (bacterial, fungal, viral), immune-mediated diseases (pemphigus foliaceus, systemic lupus erythematosus, lupoid onychodystrophy, adverse cutaneous drug reaction, vasculitis, lymphocytic plasmacytic pododermatitis), endocrinopathies (hypothyroidism, hyperadrenocorticism, diabetes mellitus), metabolic diseases (superficial necrolytic dermatitis), contact reactions, obesity, foreign bodies, and neoplasms (squamous cell carcinoma, cutaneous lymphoma) [191,192]. Dermatological signs can include variable pruritus, pain, discomfort, erythema, swelling, hypotrichosis to alopecia, erosions/ulcerations, crusts, lichenification, hyperpigmentation, interdigital papule to nodule formation, interdigital fistulation, serosanguineous to purulent exudation, paw pad hyperkeratosis, fissures and ulcerations, paronychia, and nail alterations. Secondary infections with bacteria and/or *Malassezia* are common, complicating not only the disease but also the management [14,191,192].

Due to the multiplicity of causes, a detailed history, general and dermatological examination, and basic tests such as skin scrapes, trichogram, and cytology are warranted. Bacterial culture and sensitivity testing, fungal culture, haematology and biochemistry, specific tests for infectious organisms (leishmania, distemper, and others), and histopathology may be added, depending on the suspected underlying cause [14,191,192]. 

The treatment is largely influenced by the underlying disease and should be addressed accordingly, but antiseptic shampoos, mousses, sprays, or foot soaks, as well as topical and systemic anti-inflammatory and anti-pruritic products, are often used unless contra-indicated. In cases of deep bacterial or fungal infections, additional oral antimicrobials over 8 to 12 weeks may be necessary. Severe cases with chronic, fibrotic, and hyperplastic changes may need surgical interventions or CO_2_ laser treatment. Magnesium sulphate baths (Epsom salts) can also be beneficial. Non-medical measurements such as weight reduction, soft bedding, trauma reduction, and the temporary wearing of special, waterproof dog boots are also favorable [14,173,191,195]. 

The prognosis is good to guarded, depending on how easily the underlying cause can be corrected and how chronic the changes of the paws at the timepoint of the diagnosis are [191].

#### 3.5.3. Calcinosis Circumscripta

In young dog breeds, including the Rottweiler, Boston Terrier, Boxer, and Shih Tzu, repeated trauma may cause a localized calcification of the skin called calcinosis circumscripta. In these cases, the underlying tissue as well as the calcium/phosphor homeostasis appear normal. In brachycephalic breeds, small to large, white to purple, firm, dome-shaped, sometimes ulcerated papules, nodules, or plaques filled with a chalky material often occur at the cheek and base of the ear. Cytology and histopathology are diagnostic options, and treatment is usually done by surgical excision [14,101,102,103]. 

#### 3.5.4. Dermoid Sinus/Cyst

**Figure 6:**
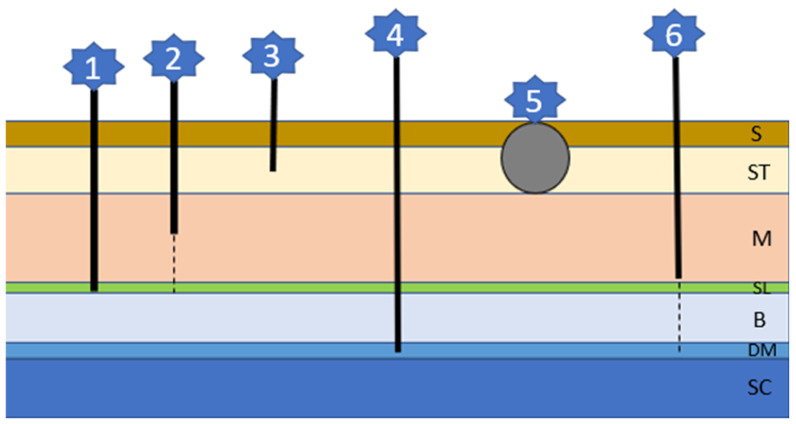
The six different sinus types of dermoid cysts (refer to text for details); S: skin; ST: Subcutaneous tissue; M: muscle; SL: supraspinous ligament; B: bone; DM: dura mater; SC: spinal cord.

This inherited problem is associated with an abnormal separation of the skin and the neural tube, leading to cyst or tube formation of different depths and lengths (Figure 6) [14,104]. Each type of cyst/tube represents involuted skin with surrounding hair follicles and glands and a lumen filled with keratin, sebum, debris, and hairs [14]. There is an association between an autosomal-dominant mutation involving fibroblast growth factors (FGF) 3, 4, and 19 being responsible for the ridge formation and oral cancer overexpressed 1 factor (ORAOV1) [196]. Although Rhodesian Ridgebacks are most commonly affected, brachycephalic breeds include Boxers, Victorian bulldogs, English Bull Terriers, French bulldogs, Shih Tzus, and Chow Chows [104,105,106,107,108,109,110,111,112,113,114]. There can be singular or multiple sinuses, mostly occurring in the cervical or thoracic region, although head involvement is described in Rottweilers [108]. Lesions are often not recognized by the owner since they occur very concealed as tufts of hair or very small openings. When secondarily infected, a fistulous wound may develop. Neurological signs occur if the defect includes the dura mater and the spinal cord and are associated with a more guarded prognosis [104,106]. A diagnosis can be made via history, clinical signs, palpation, fistulogram, myelogram, CT, or MRI. Depending on the type of sinus and possible complications, considerations between observation and conservative treatment or surgical interventions need to be made [104,106]. 

### 3.6. Other Skin Diseases

Brachycephalic breeds are predisposed to skin cyst formation and nail overgrowth, the latter especially in British bulldogs and Pugs [6,59,60]. Boxers are predisposed to gingival hyperplasia, solar dermatitis, and sternal callus [14], English bulldogs to idiopathic nasodigital hyperkeratosis [197], Boston Terriers to localized parakeratotic hyperkeratosis [198], French Mastiffs to footpad hyperkeratosis [199], and Chow Chows to post-clipping alopecia [200,201]. 

## 4. General Discussion and Ethical Considerations

Dermatological disorders are common among brachycephalic breeds. While some are a direct consequence of the anatomic abnormalities that have been selected for over generations of breeding, others are not linked to brachycephaly but highlight the consequences of small gene pool diversity within dog breeds. As breeding programs are modified to select for less extreme brachycephalic confirmation, the prevalence and expression of unrelated genetic disorders need to be carefully monitored to prevent their unwitting selection.

## 5. Conclusions

Brachycephalic dogs are not only adversely affected by their airway problems, chronic hypoxia, hypertension, sleep disorders, ophthalmologic, dental, and gastrointestinal problems, but also by lifelong dermatological dilemmas, which are often challenging to treat and negatively affect their quality of life. These are enough arguments to suggest that revised breed standards for those dogs are desperately needed.

## Figures and Tables

**Table 1:** The most common brachycephalic breeds in UK [6,13]. Breeds particularly associated with extreme brachycephaly are bolded.

Affenpinscher
**Bulldog Breeds:** Alapaha Blue Blood Bulldog; American Bulldog; British Bulldog; Bulldog; Dorset Olde Tyme Bulldogge; French Bulldog; Victorian Bulldog
Boxer; Bull Boxer; German Boxer
Brasileiro
Brussels Griffon; Griffon
**Boston Terrier**
Cavalier King Charles Spaniel
Chihuahua; Long-haired Chihuahua; short-Haired Chihuahua; Teacup Chihuahua
Chow Chow
Dogue de Bordeaux
English Toy Spaniel
Japanese Chin
Lhasa Apso
Mastiff Breeds: American Bandogge Mastiff; Bullmastiff; Cane Corso (Italian Mastiff); English Mastiff; Neapolitan Mastiff; Tibetan Mastiff
Pekingese
**Pug**
Shar Pei
Shi Tzu
Staffordshire Bull Terrier

**Table 2:** Dermatological diseases of brachycephalic breeds.

Disease Group	Disease	Breeds	References
**Congenital** **Skin Diseases**	Congenital Alopecia	Chihuahua French Bulldog Lhasa Apso	[14,15,16,17]
	Color dilution alopecia Black hair follicular dysplasia Follicular dysplasia	Blue Chow Chow Boston Terrier Boxer Cavalier King Charles Spaniel Chihuahua Shih Tzu	[14,18,19,20,21,22,23]
	Flank alopecia	Affenpinscher Boxer Chihuahua English Bulldog Staffordshire Bull Terrier	[24,25,26,27]
	Pattern baldness	Boston Terrier Boxer Chihuahua English Bulldog	[14,28]
	Ichthyosis	American Bulldog Cavalier King Charles Spaniel	[29,30,31,32,33]
	Cutaneous Asthenia	Boxer	[14,34]
	Tyrosinase deficiency	Chow Chow	[14,35]
	Caudal occipital malformation syndrome Chiari-like malformation	Affenpinscher Boston Terrier Brussels Griffon Cavalier King Charles Spaniel Chihuahua French Bulldog Pomeranian Pug Shih Tzu	[36,37,38,39,40,41,42,43]
	Primary secretory otitis media Otitis media with effusion	Cavalier King Charles Spaniel Boxer Boston Terrier	[44,45,46]
**Infectious** **Skin Diseases**	Canine Demodicosis	Boxer Boston Terrier Chihuahua Chow Chow English Bulldog French Bulldog Pugs Shih Tzu Staffordshire Bull Terrier Shar Pei	[10,47,48,49,50,51,52,53,54,55,56]
*Fungal*	*Malassezia* dermatitis	Boxer Cavalier King Charles Spaniel English Bulldog Lhasa Apso Shih Tzu	[14,57,58]
*Bacterial*	Superficial pyoderma	Boxer British Bulldog Bullmastiff Pug Shar Pei	[9,14,59,60]
	Hot spot	British Bulldogs Pugs	[9,59,60,61]
	Muzzle folliculitis and furunculosis	Boxer British Bulldog	[3,14,62]
	Canine leproid Granuloma	Boxer	[14,63,64]
*Viral*	Viral pigmented Plaques	Australian Terrier Boston Terrier Chihuahua French Bulldog Pug	[65,66,67,68]
*Mixed*	Otitis externa	Boxers British Bulldogs Pugs	[8,59,60,69]
**Immunological Diseases**	Primary immune Deficiencies	Bull Terrier Chow Chow Pomeranian Shar Pei	[14,70,71,72,73,74]
	Hypersensitivities	American Bulldog Boston Terrier Boxer Chow Chow English Bulldog French Bulldog Lhasa Apso Pug Shar Pei Shih Tzu Staffordshire Bullterrier	[14,75,76,77,78,79,80,81,82,83,84]
	Pemphigus foliaceus	Chow Chow	[85,86,87,88,89]
	Uveodermatologic Syndrome	Chow Chow	[14,90,91]
	Acute febrile vasculitis	Shar Pei	[92,93,94,95]
	Sterile granuloma and pyogranuloma syndrome	Boxer English Bulldog French Mastiff	[14,96]
**Miscellaneous** **Skin** **Diseases**	Skin fold dermatitis	Boston Terriers British Bulldog Pekingese Pug Shar Pei	[3,9,12,14,59,60,97,98,99]
	Anal sac disease	Pugs	[3,6,97,100]
	Calcinosis circumscripta	Boston Terrier Boxer Shih Tzu	[14,101,102,103]
	Dermoid sinus/cyst	Boxers Chow Chow English Bull Terrier French Bulldog Shih Tzu Victorian Bulldog	[104,105,106,107,108,109,110,111,112,113,114]

## Data Availability

Not applicable.
